# Sarcopenia and Age-Related Endocrine Function

**DOI:** 10.1155/2012/127362

**Published:** 2012-05-28

**Authors:** Kunihiro Sakuma, Akihiko Yamaguchi

**Affiliations:** ^1^Research Center for Physical Fitness, Sports and Health, Toyohashi University of Technology, 1-1 Hibarigaoka, Tenpaku-cho, Toyohashi 441-8580, Japan; ^2^School of Dentistry, Health Sciences University of Hokkaido, Kanazawa, Ishikari-Tobetsu, Hokkaido 061-0293, Japan

## Abstract

Sarcopenia, the age-related loss of skeletal muscle, is characterized by a deterioration of muscle quantity and quality leading to a gradual slowing of movement, a decline in strength and power, and an increased risk of fall-related injuries. Since sarcopenia is largely attributed to various molecular mediators affecting fiber size, mitochondrial homeostasis, and apoptosis, numerous targets exist for drug discovery. In this paper, we summarize the current understanding of the endocrine contribution to sarcopenia and provide an update on hormonal intervention to try to improve endocrine defects. Myostatin inhibition seems to be the most interesting strategy for attenuating sarcopenia other than resistance training with amino acid supplementation. Testosterone supplementation in large amounts and at low frequency improves muscle defects with aging but has several side effects. Although IGF-I is a potent regulator of muscle mass, its therapeutic use has not had a positive effect probably due to local IGF-I resistance. Treatment with ghrelin may ameliorate the muscle atrophy elicited by age-dependent decreases in growth hormone. Ghrelin is an interesting candidate because it is orally active, avoiding the need for injections. A more comprehensive knowledge of vitamin-D-related mechanisms is needed to utilize this nutrient to prevent sarcopenia.

## 1. Introduction

Age-related declines in muscle mass and strength, known as sarcopenia, are often an important antecedent of the onset of disability in older adulthood. Although the term is applied clinically to denote loss of muscle mass, sarcopenia is often used to describe both a set of cellular processes (denervation, mitochondrial dysfunction, and inflammatory and hormonal changes) and a set of outcomes such as decreased muscle strength, decreased mobility and function [1], increased fatigue, a greater risk of falls [2], and reduced energy needs. Lean muscle mass generally contributes up to ~50% of total body weight in young adults but declines with aging to be 25% at 75–80 years old [3, 4]. The loss of muscle mass is typically offset by gains in fat mass. The loss is most notable in the lower limb muscle groups, with the cross-sectional area of the vastus lateralis being reduced by as much as 40% between the ages of 20 and 80 yr [5].

 Several possible mechanisms for age-related muscle atrophy have been described; however, the precise contribution of each is unknown. Age-related muscle loss is a result of reductions in the size and number of muscle fibers [6] possibly due to a multifactorial process that involves physical activity, nutritional intake, oxidative stress, and hormonal changes [2, 7]. The specific contribution of each of these factors is unknown, but there is emerging evidence that the disruption of several positive regulators (Akt and serum response factor) of muscle hypertrophy with age is an important feature in the progression of sarcopenia [8–10]. In contrast, many investigators have failed to demonstrate an age-related enhancement in levels of common negative regulators (Atrogin-1, myostatin, and calpain) in senescent mammalian muscles.

 Several lines of evidence point to inflammation being associated with loss of muscle strength and mass with aging [11]. Animal studies have shown that the administration of interleukin (IL)-6 or tumor necrosis factor (TNF)-*α* increases skeletal muscle breakdown, decreases the rate of protein synthesis, and reduces plasma concentrations of insulin-like growth factor [12, 13]. In older men and women, higher levels of IL-6 and C-reactive protein (CRP) were associated with a two- to threefold greater risk of losing more than 40% of grip strength over 3 years [14]. On the other hand, several studies have indicated age-related endocrine defects such as decreases in anabolic hormones (testosterone, estrogen, growth hormone (GH), and insulin-like growth factor-I (IGF-I)) [15–18]. Although hormonal supplementation for the elderly has been conducted on a large scale, it was found not to be effective against sarcopenia and to have minor side effects [9, 10, 15, 16, 19, 20]. In this paper, we summarize the current understanding of the endocrine contribution to sarcopenia and provide an update on practical hormonal intervention for the elderly.

## 2. The Adaptative Changes in Catabolic Mediators

### 2.1. TNF-*α*

 Inflammation may negatively influence skeletal muscle through direct catabolic effects or through indirect mechanisms (i.e., decreases in GH and IGF-I concentrations, induction of anorexia, etc.) [21]. There is growing evidence that higher levels of inflammatory markers are associated with physical decline in older individuals, possibly through the catabolic effects of these markers on muscle. In an observational study of more than 2000 men and women, TNF-*α* showed a consistent association with declines in muscle mass and strength [22]. The impact of inflammation on the development of sarcopenia is further supported by a recently published animal study showing that a reduction in low-grade inflammation by ibuprofen in old (20 months) rats resulted in a significant decrease in muscle mass loss [23]. An age-related disruption of the intracellular redox balance appears to be a primary causal factor for a chronic state of low-grade inflammation. More recently, Chung et al. [24] hypothesized that abundant nuclear factor-*κ*B (NF-*κ*B) protein induced age-related increases in IL-6 and TNF-*α*. Moreover, reactive oxygen species (ROS) also appear to function as second messengers for TNF-*α* in skeletal muscle, activating NF-*κ*B either directly or indirectly [25]. Indeed, marked production of ROS has been documented in muscle of the elderly [26, 27]. However, it is not clear whether NF-*κ*B signaling is enhanced with age. Despite some evidence supporting enhanced NF-*κ*B signaling in type I fibers of aged skeletal muscle, direct evidence for increased activation and DNA binding of NF-*κ*B is lacking [28, 29]. For example, Phillips and Leeuwenburgh [29] found that neither p65 protein expression nor the binding activity of NF-*κ*B was significantly altered in the vastus lateralis muscles of 26-month-old rats despite the marked upregulation of TNF-*α* expression in both blood and muscle. Upregulated TNF-*α* expression in serum and muscle seems to enhance apoptosis through increased mitochondrial defects resulting in a loss of muscle fibers [29–31]. It has been shown that TNF-*α* is one of the primary signals inducing apoptosis in muscle.

### 2.2. Myostatin

 Myostatin was first discovered during screening for new members of the transforming growth factor-*β* (TGF-*β*) superfamily and shown to be a potent negative regulator of muscle growth [32]. Like other family members, myostatin is synthesized as a precursor protein that is cleaved by furin proteases to generate the active C-terminal dimer. Most, if not all, of the myostatin protein that circulates in blood also appears to exist in an inactive complex with a variety of proteins, including the propeptide [33]. The latent form of myostatin seems to be activated *in vitro* by dissociation from the complex with either acid or heat treatment [33, 34] or by proteolytic cleavage of the propeptide with members of the bone morphogenetic protein-1/tolloid family of metalloproteases [34].

 Studies indicate that myostatin inhibits cell cycle progression and reduces levels of myogenic regulatory factors (MRFs), thereby controlling myoblastic proliferation and differentiation during developmental myogenesis [35–37]. Myostatin binds to and signals through a combination of ActRIIA/B receptors on the cell membrane but has higher affinity for ActRIIB. On binding to ActRIIB, myostatin forms a complex with either activin receptor-like kinase (ALK) 4 or ALK5 to activate (phosphorylate) the Smad2/3 transcription factors. Then Smad2/3 are translocated and modulate the nuclear transcription of genes such as MyoD [38] via a TGF-*β*-like mechanism. More recently, the IGF-I-Akt-mTOR (mammalian target of rapamycin) pathway, which mediates both differentiation in myoblasts and hypertrophy in myotubes, has been shown to inhibit myostatin-dependent signaling. Blockade of the Akt-mTOR pathway, using siRNA to RAPTOR, a component of TORC1 (TOR signaling complex 1), facilitates myostatin's inhibition of muscle differentiation because of an increase in Smad2 phosphorylation [39]. In contrast, Smad2/3 inhibition promotes muscle hypertrophy partially dependent on mTOR signaling [40].

 Several researchers have investigated the effect of inhibiting myostatin to counteract sarcopenia using animals [41, 42]. Lebrasseur et al. [41] found that treatment with a mouse chimera of antihuman myostatin antibody (24 mg/Kg, 4 weeks), a drug for inhibiting myostatin, elicited a significant increase in muscle mass and in running performance probably due to decreased levels of phosphorylated Smad3 and Muscle ring finger-1 (MuRF-1). More recently, Murphy et al. [42] showed, by way of once weekly injections, that a lower dose of this anti-human myostatin antibody (10 mg/Kg) significantly increased the fiber cross-sectional area (by 12%) and *in situ* muscle force (by 35%) of aged mice (21 mo old). These findings highlight the therapeutic potential of antibody-directed myostatin inhibition for sarcopenia by inhibiting protein degradation. Although many researchers expect myostatin levels to be increased not only in muscle but also in serum, blood myostatin levels have not been shown to increase with age [43].

### 2.3. Glucocorticoid

 Glucocorticoid-associated atrophy appears to be specific to type II or phasic muscle fibers. In a study of controlled hypercortisolaemia in healthy men [44], experimental inactivity increased the catabolic effect of glucocorticoids, suggesting that an absence of mechanical signals potentiates the effect. The mechanism of glucocorticoid-induced atrophy may involve upregulated expression of myostatin and glutamine synthetase, the latter via the glucocorticoid receptor's interaction with the glutamine synthetase promoter [45]. Glucocorticoids inhibit the physiological secretion of GH and appear to induce IGF-I activity in target organs. Changes in steroid-induced glutamine synthetase represent a potential mechanism of action, and dose-dependent inhibition of glutamine synthetase by IGF-I was observed in rat L6 cells [46].

 The increased incidence of various diseased states during aging is associated with the hypersecretion of glucocorticoids [47, 48]. In addition, when adult (7-month-old) and aged (22-month-old) rats received dexamethasone (approximately 500 *μ*g/Kg body weight/day) in their drinking water for 5-6 days, muscle wasting was much more rapid in aged animals [47]. Furthermore, glucocorticoids induced prolonged leucine resistance to muscle protein synthesis in old rats [49]. Still, it remains to be directly elucidated, using pharmacological inhibitors for glucocorticoids, whether age-related increases in serum glucocorticoid levels actually inhibit protein synthesis and/or enhance protein degradation.

### 2.4. Interleukin-6 and CRP

 IL-6 and CRP, known as “geriatric cytokines,” are multifunctional cytokine produced in situations of trauma, stress, and infection. During the aging process, levels of both IL-6 and CRP in plasma become elevated. The natural production of cytokines is likely beneficial during inflammation, but overproduction and the maintaining of an inflammatory state for long periods of time, as seen in elderly individuals, are detrimental [50, 51]. A number of authors have demonstrated that a rise in plasma levels of proinflammatory cytokines, especially IL-6, and proteins under acute conditions is associated with a reduction in mobility as well as a reduced capacity to perform daily activities, the development of fragility syndrome, and increased mortality rates [50–52]. In older men and women, higher levels of IL-6 and CRP were associated with a two- to threefold greater risk of losing more than 40% of grip strength over 3 years [14]. In contrast, there were no longitudinal associations between inflammatory markers and changes in grip strength among high functioning elderly participants from the MacArthur Study of Successful Ageing [53]. More recently, Hamer and Molloy [54] demonstrated, in a large representative community-based cohort of older adults (1,926 men and 2,260 women (aged 65.3 ± 9.0 years)), that CRP was associated with poorer hand grip strength and chair stand performance in women but only chair stand performance in men. In addition, Haddad et al. [55] demonstrated atrophy in the tibialis anterior muscle of mice following the injection of relatively low doses of IL-6.

 In a recent randomized trial that employed aerobic and strength training in a group of elderly participants, significant reductions in various inflammatory markers (IL-6, CRP, and IL-18) were observed for aerobic but not strength training [56]. In contrast, combined resistance and aerobic training that increased strength by 38% resulted in significant reductions in CRP [57]. More descriptive data appears to be needed whether IL-6 and CRP have an actual catabolic effect in sarcopenic muscle.

## 3. Anabolic Hormones in Sarcopenic Muscle

### 3.1. Testosterone

 In males, levels of testosterone decrease by 1% per year, and those of bioavailable testosterone by 2% per year from age 30 [16, 58, 59]. In women, testosterone levels drop rapidly from 20 to 45 years of age [60]. Testosterone increases muscle protein synthesis [61], and its effects on muscle are modulated by several factors including genetic background, nutrition, and exercise [62].

 Numerous studies of treatment with testosterone in the elderly have been performed over the past few years [63–66]. In 1999, Snyder et al. [66] suggested that increasing the level of testosterone in old men to that seen in young men increased muscle mass but did not result in functional gains in strength. Systemic reviews of the literature [67] have concluded that testosterone supplementation attenuates several sarcopenic symptoms including decreases in muscle mass [64–66] and grip strength [63]. For instance, a recent study of 6 months of supraphysiological dosage of testosterone in a randomized placebo-controlled trial reported increased leg lean body mass and leg and arm strength [68]. Although there are significant increases in strength among elderly males given high doses of testosterone, the potential risks may outweigh the benefits. Risks associated with testosterone therapy in older men include sleep apnea, thrombotic complications, and the increased risk of prostate cancer [69].

 These side effects have driven the necessity for drugs that demonstrate improved therapeutic profiles. Novel, nonsteroidal compounds, called selective androgen receptor modulators, have shown tissue-selective activity and improved pharmacokinetic properties. Whether these drugs are effective in treating sarcopenia has yet to be shown [70]. Dehydroepiandrosterone (DHEA) is marketed as a nutritional supplement in the USA and is available over the counter. Unlike testosterone and estrogen, DHEA is a hormone precursor which is converted into sex hormones in specific target tissues [71]. However, supplementation of DHEA in aged men and women resulted in an increase in bone density and testosterone and estradiol levels, but no changes in muscle size, strength, or function [72, 73].

### 3.2. Estrogen

 It has been hypothesized that menopause transition and the subsequent decline in estrogen may play a role in muscle mass loss [7, 18]. Van Geel et al. [74] reported a positive relationship between lean body mass and estrogen levels. Similarly, Iannuzzi-Sucich et al. [75] observed that muscle mass is correlated significantly with plasma estrone and estradiol levels in women. However, Baumgartner et al. [76] reported that estrogen levels were not associated with muscle mass in women aged 65 years and older. The mechanisms by which decrease in estrogen levels may have a negative effect on muscle mass are not well understood but may be associated with an increase in proinflammatory cytokines, such as TNF-*α* and IL-6, which might be implicated in the apparition of sarcopenia [77]. Furthermore, estrogen could have a direct effect on muscle mass since it has been shown that skeletal muscle has estrogen beta-receptors on the cell membrane [78]. Therefore, a direct potential mechanistic link could exist between low estrogen levels and a decrease in protein synthesis. Further studies are needed to investigate this hypothesis. Nevertheless, before reaching a conclusion on the contribution of estrogens to the onset of sarcopenia, it would be important to measure urinary estrogen metabolites since a relationship between breast cancer and urinary estrogen metabolites has been shown [79].

### 3.3. GH

 Growth hormone (GH) is a single-chain peptide of 191 amino acids produced and secreted mainly by the somatotrophs of the anterior pituitary gland. GH coordinates the postnatal growth of multiple target tissues, including skeletal muscle [80]. GH secretion occurs in a pulsatile manner with a major surge at the onset of slow-wave sleep and less conspicuous secretory episodes a few hours after meals [81] and is controlled by the actions of two hypothalamic factors, GH-releasing hormone (GHRH), which stimulates GH secretion, and somatostatin, which inhibits GH secretion [82]. The secretion of GH is maximal at puberty accompanied by very high circulating IGF-I levels [83], with a gradual decline during adulthood. Indeed, circulating GH levels decline progressively after 30 years of age at a rate of ~1% per year [84]. In aged men, daily GH secretion is 5- to 20-fold lower than that in young adults [85]. The age-dependent decline in GH secretion is secondary to a decrease in GHRH and to an increase in somatostatin secretion [86].

 With respect to the somatomedin hypothesis, the growth-promoting actions of GH are mediated by circulating or locally produced IGF-I [87]. GH-induced muscle growth may be mediated in an endocrine manner by circulating IGF-I derived from liver and/or in an autocrine/paracrine manner by direct expression of IGF-I from target muscle via GH receptors on muscle membranes. The effects of GH administration on muscle mass, strength and physical performance are still under debate [19]. In animal models, GH treatment is very effective at inhibiting sarcopenic symptoms such as muscle atrophy and decreases in protein synthesis particularly in combination with exercise training [88]. The effect of GH treatment for elderly subjects is controversial. Some groups demonstrated an improvement in strength after long-term administration (3–11 months) of GH [89]. In contrast, many researchers have found that muscle strength or muscle mass did not improve on supplementation with GH [19, 89]. One recent study reported a positive effect for counteracting sarcopenia after the administration of both GH and testosterone [90]. Several reasons may underlie the ineffectiveness of GH treatment in improving muscle mass and strength in the elderly, such as a failure of exogeneous GH to mimic the pulsatile pattern of natural GH secretion or the induction of GH-related insulin resistance. In addition, reduced mRNA levels of the GH receptor in skeletal muscle have been observed in older versus younger healthy men, exhibiting a significant negative relationship with myostatin levels [91]. It should also be considered that the majority of the trials conducted on GH supplementation have reported a high incidence of side effects, including soft tissue edema, carpal tunnel syndrome, arthralgias, and gynecomastica, which pose serious concerns especially in older adults. Therefore, one should pay very careful attention when administering GH to the elderly.

 There is evidence that the age-associated decline in GH levels in combination with lower IGF-I levels contributes to the development of sarcopenia [92]. IGF-I is perhaps the most important mediator of muscle growth and repair [93] possibly by utilizing Akt-mTOR-p70S6K (p70 ribosomal protein S6 kinase) signaling. Although the transgenic approach of upregulating IGF-I expression in skeletal muscle would be appropriate for inhibiting sarcopenia, the administration of IGF-I to the elderly has resulted in controversial findings on muscle strength and function [94]. The ineffectiveness may be attributable to age-related insulin resistance to amino acid transport and protein synthesis [95] or a marked decrease in IGF-I receptors [96, 97] and receptor affinity for IGF-I [98] in muscle with age. Wilkes et al. [99] demonstrated a reduced effect of insulin on protein breakdown in the legs in older versus younger subjects probably due to the blunted activation of Akt by insulin. More comprehensive reviews on insulin resistance in sarcopenia can be found elsewhere [95].

### 3.4. Ghrelin

**Figure 1:**
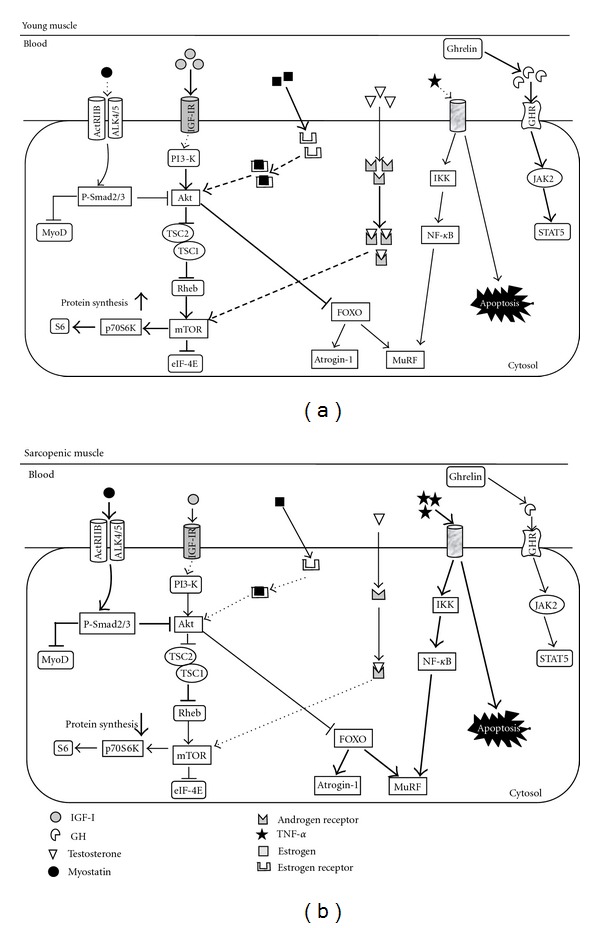
(a) In young muscle, abundant serum IGF-I can stimulate protein synthesis by activating Akt/mTOR/p70S6K pathway. Akt blocks the nuclear translocation of FOXO to inhibit the expression of Atrogin-1 and MuRF and the consequent protein degradation. Abundant serum GH, which is induced by ghrelin, activates JAK2-STAT5 signaling to promote muscle-specific gene transcription necessary to hypertrophy. In young muscle, testosterone and estrogen bind these intramuscular receptors (androgen receptor and estrogen receptor (*α* and *β*)), and activate mTOR and Akt, respectively. Lower serum amount of myostatin and TNF-*α* failed to activate signaling candidates (Smad 2/3, NF-*κ*B, etc.) enhancing protein degradation. (b) In sarcopenic muscle, myostatin signals through the activin receptor IIB (ActRIIB), ALK4/5 heterodimer seems to activate Smad2/3 and blocking of MyoD transactivation in an autoregulatory feedback loop. Abundant activated Smad2/3 inhibit protein synthesis probably due to blocking the functional role of Akt. The increased blood TNF-*α* elevates the protein degradation through IKK/NF-*κ*B signaling and enhance an apoptosis. Lower serum amount of IGF-I, GH, and anabolic hormones (testosterone and estrogen) failed to activate signaling candidates (Akt, mTOR, STAT5, etc.) enhancing protein synthesis. The impaired regulation of FOXO by Akt results in abundant expression of Atrogin-1 and MuRF and the consequent protein degradation in sarcopenic muscle.

 Ghrelin is a 28-amino-acid peptide mainly produced by cells in the stomach, intestines, and hypothalamus [100]. Ghrelin is a natural ligand for the GH-secretagogue receptor (GHS-R), which possesses a unique fatty acid modification, an n-octanoylation, at Ser 3 [101]. Ghrelin plays a critical role in a variety of physiological processes, including the stimulation of GH secretion and regulation of energy homeostasis by stimulating food intake and promoting adiposity via a GH-independent mechanism [100]. In contrast, ghrelin inhibits the production of anorectic proinflammatory cytokines, including IL-1*β*, IL-6, and TNF-*α* [102]. Because of their combined anabolic effects on skeletal muscle and appetite, ghrelin and low-molecular-weight agonists of the ghrelin receptor are considered attractive candidates for the treatment of cachexia [103]. For example, Nagaya et al. [104] gave human ghrelin (2 *μ*g/Kg twice daily intravenously) for 3 weeks to cachexic patients with chronic obstructive pulmonary disease in an open-label study. After ghrelin therapy, significant increases from baseline measurements were observed for body weight, lean body mass, food intake, hand grip strength, maximal inspiratory pressure, and Karnofsky performance score [104]. In another unblinded study, the same group demonstrated that treatment with human ghrelin (2 *μ*g/Kg twice daily intravenously, 3 weeks) significantly improved several parameters (eg., lean body mass measured by dual-energy X-ray absorption and left ventricular ejection fraction) in 10 patients with chronic heart failure [105]. In a 1-year placebo-controlled study in healthy older adults over the age of 60 years given an oral ghrelin-mimetic (MK-677), an increase in appetite was observed [106]. The study did not show a significant increase in strength or function in the ghrelin-mimetic treatment group, when compared to the placebo group; however, a tendency was observed [106]. As pointed out in a recent review by Nass et al. [20], the use of this compound induces the potential deterioration of insulin sensitivity and development of diabetes mellitus in older adults with impaired glucose tolerance.  Figure 1 provides an overview of several regulators for muscle mass in both young and sarcopenic mammalian muscles.

### 3.5. Vitamin D

 Vitamin D has been traditionally considered a key regulator of bone metabolism and calcium and phosphorus homeostasis through negative feedback with the parathyroid hormone [107, 108]. It is also well established that vitamin D deficiency causes rickets in children and osteomalacia and osteoporosis in adults. A large and growing body of evidence suggests that vitamin D is not only necessary for bone tissue and calcium metabolism but may also represent a crucial determinant for the development of major (sub)clinical conditions and health-related events [107, 109].

 Today, approximately 1 billion, mostly elderly people, worldwide have vitamin D deficiency. The prevalence of low vitamin D concentrations in subjects older than 65 years of age has been estimated at approximately 50% [110–112], but this figure is highly variable because it is influenced by sociodemographic, clinical, therapeutic, and environmental factors. Similarly there is an age-dependent reduction in vitamin D receptor expression in skeletal muscle [113]. Prolonged vitamin D deficiency has been associated with severe muscle weakness, which improves with vitamin D supplementation [114]. The histological examination of muscle tissue from subjects with osteomalacia is characterized by increased interfibrillar space, intramuscular adipose tissue infiltrates, and fibrosis [115]. Interestingly, muscle biopsies performed before and after vitamin D supplementation have documented an increased number and sectional area of type II (or fast) muscle fibers [113, 116].

 A large body of evidence currently demonstrates that low vitamin D concentrations represent an independent risk factor for falls in the elderly [117–119]. Supplementation with vitamin D in double-blind randomized-controlled trials has been shown to increase muscle strength and performance and reduce the risk of falling in community-living elderly and nursing home residents with low vitamin D levels [120–124]. In contrast, several groups found no positive effect of vitamin D supplementation on fall event outcomes [125–127]. Cesari et al. [128] attributed these contradictory findings to the selection criteria adopted to recruit study populations, adherence to the intervention, or the extreme heterogeneity of cut-points defining the status of deficiency. A more comprehensive knowledge on vitamin-D-related mechanisms may provide a very useful tool preventing muscle atrophy for older persons (sarcopenia).

## 4. Conclusion

 Given the current and future demographic age shift in the world's population, intense research in this area is imperative. Decreases in muscle mass have been shown to be a key element in the development of frailty. Currently, resistance training combined with amino-acid-containing supplements would be the best way to prevent age-related muscle wasting and weakness. Comprehensive trials have demonstrated that supplementation with GH, IGF-I, or estrogen has a minor sarcopenia-inhibiting effect. Testosterone supplementation in large amounts improves muscle defects with aging but has several side effects. Ghrelin-mimetics which have the ability to increase caloric intake as well as to increase lean body mass in the older population could be potentially beneficial and reverse the catabolic state associated with sarcopenia. Myostatin inhibition seems to be an intriguing strategy for attenuating sarcopenia as well as muscular dystrophy.

## Abbreviations

ActRIIB: Activin type IIB receptor
ALK: Activin receptor-like kinase
CRP: C-reactive protein
DHEA: Dehydroepiandrosterone
GH: Growth hormone
GHRH: Growth hormone releasing hormone
IGF-I: Insulin-like growth factor-I
IL: Interleukin
mTOR: Mammalian target of rapamycin
MuRF-1: Muscle ring finger-1
NF-*κ*B: Nuclear factor-kappaB
p70S6K: p70 ribosomal protein S6 kinase
ROS: Reactive oxygen species
TNF-*α*: Tumor necrosis factor-*α*
TORC1: TOR signaling complex 1.
